# Comparative pharmacovigilance analysis of suicidality-related adverse events among GLP-1 and non-GLP-1 anti-obesity drugs in the FDA Adverse Event Reporting System

**DOI:** 10.1007/s11096-026-02099-y

**Published:** 2026-02-25

**Authors:** Jose Seijas-Amigo, Ángel Salgado-Barreira, Diego Rodriguez-Penas, Begoña Cardeso-Paredes, Marta Ribeiro-Ferreiro, Moisés Rodriguez-Mañero, Jose Ramon Gonzalez-Juanatey

**Affiliations:** 1 Cardiology Department, Complejo Hospitalario Universidad de Santiago de Compostela, Santiago de Compostela, Spain; 2 Instituto de Investigación Sanitaria de Santiago de Compostela (FIDIS), Santiago, Spain; 3 Centro de Investigación Biomédica en Red de Enfermedades Cardiovasculares (CIBERCV), Madrid, Spain; 4 Department of Preventive Medicine and Public Health, University of Santiago de Compostela, Santiago de Compostela, Spain; 5 Consortium for Biomedical Research in Epidemiology and Public Health (CIBER en Epidemiología y Salud Pública CIBERESP), Carlos III Health Institute, Madrid, Spain; 6 University of Santiago de Compostela, Santiago de Compostela, Spain

**Keywords:** Adverse drug reactions, Anti-obesity drugs, FAERS, GLP-1 receptor agonists, Pharmacovigilance, Suicidality

## Abstract

**Introduction:**

Regulatory reviews in 2023–2024 reignited concern about possible suicidality with Glucagon-like peptide-1 (GLP-1) receptor agonists used for weight management. While clinical trials and real-world studies have not confirmed an increased risk, comparative post-marketing analyses across all anti-obesity agents are scarce.

**Aim:**

To compare disproportional reporting of suicidality-related adverse events among GLP-1/dual incretin versus non-GLP-1 anti-obesity drugs in the FDA Adverse Event Reporting System (FAERS).

**Method:**

We conducted a retrospective disproportionality study using FAERS (January 2012–February 2025). Reports submitted from the United States with the study drug listed as primary suspect were retrieved via openFDA. Suicidality terms were predefined (MedDRA Preferred Terms: suicidal ideation, suicide attempt, completed suicide). Reporting Odds Ratios (RORs) with 95% confidence intervals (CIs) were calculated for each drug and at class level (GLP-1/dual incretin vs non-GLP-1). Haldane–Anscombe corrections were applied where needed.

**Results:**

Among approximately 78,000 anti-obesity reports, 207 (approximately 0.3%) involved suicidality-related events. For semaglutide, RORs were 1.39 (95% CI 0.99–1.94) for suicidal ideation, 1.38 (0.46–4.15) for suicide attempt, and 1.72 (0.62–4.74) for completed suicide, none statistically significant. Liraglutide showed ROR 1.01 (0.66–1.55) for suicidal ideation and 18.11 (6.96–47.15) for completed suicide based on 14 cases. Tirzepatide yielded RORs below unity for all outcomes. Naltrexone/bupropion showed elevated disproportional reporting for suicidal ideation (ROR 3.84; 95% CI 2.89–5.12) and suicide attempt (ROR 4.11; 95% CI 1.62–10.45.

**Conclusion:**

GLP-1 and dual-incretin agents did not show disproportionality signals for suicidal ideation or suicide attempt. A statistically significant disproportionality signal for completed suicide was observed for liraglutide; however, this estimate was based on few cases and displayed wide confidence intervals, warranting cautious interpretation. These findings support an overall neutral psychiatric safety profile for incretin-based therapies while underscoring the need for continued monitoring of rare events such as completed suicide.

**Supplementary Information:**

The online version contains supplementary material available at 10.1007/s11096-026-02099-y.

## Impact statements

- Patients: Incretin-based weight-loss drugs did not show increased reporting of suicidality versus other anti-obesity medicines in FAERS.
- Pharmacy/Clinical practice: Pharmacists can reassure patients while continuing routine monitoring, particularly when using non-GLP-1 combinations such as naltrexone/bupropion.
- Healthcare systems: Comparative signal assessment supports targeted pharmacovigilance rather than class-wide restrictions for GLP-1/dual incretin therapies.

## Introduction

The global prevalence of obesity has more than tripled over the past five decades, and pharmacological therapy has become an essential adjunct to lifestyle modification for long-term weight management [1, 2]. Glucagon-like peptide-1 receptor agonists (GLP-1 RAs) such as liraglutide and semaglutide, and more recently dual GLP-1/GIP agonists like tirzepatide, have demonstrated substantial weight loss and cardiometabolic benefit in randomized clinical trials [3, 4]. Non-GLP-1 combinations, naltrexone/bupropion and phentermine/topiramate, remain alternative options approved by the U.S. Food and Drug Administration (FDA) for chronic weight management [5].

In 2023, the European Medicines Agency (EMA) and the FDA announced formal evaluations of post-marketing reports describing suicidal thoughts or self-injury in individuals treated with GLP-1 RAs for obesity [6, 7]. These alerts generated extensive media coverage and a subsequent surge in pharmacovigilance reporting, emphasizing the need for systematic signal verification. Although neuropsychiatric events such as depression and suicidality were not increased in pivotal trials of liraglutide or semaglutide (STEP program) [8–10], spontaneous reporting systems remain valuable for detecting rare or unanticipated safety issues that may not surface during controlled studies.

Recent analyses of the FDA Adverse Event Reporting System (FAERS) and other international databases have yielded mixed findings. Wang et al. (2025) conducted a disproportionality analysis restricted to semaglutide, liraglutide, and tirzepatide (2004–2024) and observed significant reporting signals for semaglutide in depression (Reporting Odds Ratio (ROR) 1.87) and suicide/self-injury (ROR 1.73), but not for the other agents [11]. Guirguis et al. (2024) identified similar trends using FAERS with metformin and orlistat as comparators, though the authors noted strong influence of notoriety bias and regional reporting practices [12]. In contrast, McIntyre et al. (2025) replicated the analysis in VigiBase®, the World Health Organization (WHO) pharmacovigilance repository, and did not confirm a robust signal, suggesting that the FAERS observations may reflect differential reporting rather than causal association [13].

Complementary real-world evidence (RWE) and clinical trial data further challenge a causal link. A nationwide electronic-health-record study published in 2024 found semaglutide associated with lower risk of suicidal ideation compared with other anti-obesity or antidiabetic therapies [14]. Likewise, a claims-based analysis in 2025 reported no excess incidence of suicidality among semaglutide-treated adults [15]. In the placebo-controlled STEP 1–5 trials, rates of suicidal ideation were ≤ 1% and comparable between treatment and control groups [8]. Collectively, these findings suggest that post-marketing signals may not translate into clinically meaningful risk.

Nevertheless, existing pharmacovigilance studies have largely focused on GLP-1 RAs alone, omitting other FDA-approved anti-obesity medications that act through distinct neurochemical pathways. Bupropion-containing regimens, for instance, carry theoretical neuropsychiatric implications due to dopaminergic activity, and recent post-marketing data suggest possible isolated safety signals, warranting continued monitoring [16]. Similarly, phentermine/topiramate has been associated with anxiety or insomnia but not suicidality [17]. Evaluating all weight-loss agents within a unified framework is therefore essential to determine whether the observed signals are molecule-specific or reflect broader class effects and reporting dynamics.

The present study was designed to provide an updated comparative pharmacovigilance analysis of suicidality-related adverse events across all FDA-approved anti-obesity drugs from 2012 to 2025 using FAERS. By incorporating both incretin-based and non-incretin medications and restricting to U.S. primary suspect reports, this analysis aims to contextualize previous molecule-focused findings, assess class-wide consistency after the 2023–2024 regulatory alerts, and determine whether any agent demonstrates disproportionate reporting relative to its peers. Establishing the absence or presence of a class-level signal is critical for clinicians, regulators, and patients amid the rapidly expanding use of anti-obesity pharmacotherapy.

### Aim

To compare disproportional reporting of suicidality-related adverse events among GLP-1/dual incretin versus non-GLP-1 anti-obesity drugs in the FDA Adverse Event Reporting System.

## Method

### Data source and study design

We performed a retrospective pharmacovigilance study using data from the U.S. Food and Drug Administration Adverse Event Reporting System (FAERS), a spontaneous reporting database collecting adverse drug reaction (ADR) notifications from healthcare professionals, consumers, and manufacturers worldwide. Reports from January 1, 2012, through February 28, 2025, were extracted via the openFDA application programming interface (API; https://open.fda.gov).

The analysis followed recommendations from the European Medicines Agency (EMA) and the International Society of Pharmacovigilance for disproportionality analyses [18–20].

### Selection of anti-obesity drugs

All FDA-approved anti-obesity medications indicated for chronic weight management were included:

- GLP-1 receptor agonists: liraglutide (Saxenda®), semaglutide (Wegovy®)
- Dual GLP-1/GIP receptor agonist: tirzepatide (Zepbound®)
- Non-GLP-1 combinations: naltrexone/bupropion (Contrave®) and phentermine/topiramate (Qsymia®)

Setmelanotide (Imcivree®) was excluded due to its orphan indication for genetic obesity syndromes, which involves a distinct patient population and safety profile. Reports were restricted to those submitted from the United States and identifying the study drug as the primary suspect (*patient. drug. Drug characterization* = *1*) to reduce confounding by concomitant therapies [21]. Duplicate cases were automatically removed by FAERS based on unique case identifiers.

These restrictions were applied to enhance internal validity. Limiting analyses to U.S. reports reduces heterogeneity in MedDRA coding practices, reporting completeness, and submission workflows across regions. Similarly, restricting to primary suspect drugs minimizes attribution noise when multiple drugs are listed in the same report, thereby improving the specificity of the signal detection process.

### Event identification

Suicidality-related adverse events were defined according to MedDRA version 27.0, encompassing the following Preferred Terms (PTs): suicidal ideation, suicide attempt, and completed suicide. Each PT was analyzed separately to avoid aggregation bias, following the approach recommended in previous FAERS-based suicidality studies [11, 22].

Suicidality-related adverse events were retrieved at the level of MedDRA Preferred Terms (PTs). All FAERS reports mapped to these PTs, irrespective of the underlying Lowest Level Terms (LLTs), were included in the analysis. A list of the suicidality-related PTs and representative LLTs used in our queries is provided in Supplementary Table S1.

Given the markedly unequal post-marketing exposure between agents—particularly for tirzepatide, which entered the obesity market in late 2023—we anticipated substantial variability in cumulative report volume. This imbalance limits the interpretability of disproportionality estimates for recently approved drugs, where low case counts produce wide confidence intervals and immature pharmacovigilance signals.

### Comparators and analysis framework

A within-class and between-class comparative disproportionality design was used. For each anti-obesity drug, the frequency of suicidality-related reports was compared with the combined frequency of the same events reported for the remaining agents within the same therapeutic class. In addition, aggregated comparisons were performed between incretin-based (GLP-1 receptor agonists and dual GLP-1/GIP agonists) and non-GLP-1 anti-obesity drugs to explore potential class-level differences. Counts were obtained directly from open FDA queries using specific MedDRA filters and manually verified for plausibility. Analyses were restricted to reports originating from the United States to ensure data homogeneity and minimize heterogeneity related to regional reporting practices.

### Statistical analysis

Reporting Odds Ratios (RORs) and 95% confidence intervals (95% CIs) were calculated to quantify disproportionality between the target drug and its comparators. When zero counts were present in any cell, the Haldane–Anscombe correction was applied to stabilize the variance [23].

A disproportionality signal was considered statistically significant when the lower bound of the 95% CI exceeded 1.0, consistent with prior pharmacovigilance standards [18, 24].

All statistical calculations were performed in Microsoft Excel (Microsoft Corp., Redmond, WA), and the results were cross validated with outputs from OpenVigil 2.1 [25].

The ROR reflects disproportionality in reporting and should not be interpreted as a measure of incidence, absolute risk, or causality.

### Ethics approval

FAERS data are publicly available and de-identified. Therefore, this study did not require institutional review board approval or patient consent [26].

## Results

### Descriptive overview

Between 2012 and 2025, a total of approximately 78,000 FAERS reports involving FDA-approved anti-obesity drugs were retrieved, of which less than 0.5% included suicidality-related Preferred Terms (PTs), such as suicidal ideation, suicide attempt, or completed suicide. Most reports originated from the United States and were submitted after 2021, coinciding with the approval and widespread use of semaglutide (Wegovy®) for weight management.

The distribution by agent was as follows: tirzepatide (Zepbound®; approximately 23,000 reports), semaglutide (Wegovy®; approximately 20,000), liraglutide (Saxenda®; approximately 5,000), naltrexone/bupropion (Contrave®; approximately 6,000), and phentermine/topiramate (Qsymia®; approximately 5,000).

### Disproportionality analysis

As shown in Table 1, the updated disproportionality analysis revealed distinct patterns across anti-obesity agents.

**Table 1:** Reporting odds ratios (RORs) and 95% confidence intervals (CIs) for suicidality-related adverse events reported in FAERS (2012–2025) by individual anti-obesity drug

		Drug cases/total cases	Drug AE reports/Total AE reports	ROR	95%CI
Semaglutide (Wegovy®)	Suicidal ideation	44/164	7728/39,898	1.39	0.99–1.94
	Suicide attempt	4/15	7728/39,898	1.38	0.46–4.15
	Completed suicide	5/15	7728/39,898	1.72	0.62–4.74
Liraglutide (Saxenda®)	Suicidal ideation	24/184	5441/42,130	1.01	0.66–1.55
	Suicide attempt	2/17	5441/42,130	0.91	0.21–3.94
	Completed suicide	14/6	5441/42,130	18.11	6.96–47.15
Tirzepatide (Zepbound®)	Suicidal ideation	32/176	23,189/24,378	0.19	0.13–0.28
	Suicide attempt	2/17	23,189/24,378	0.12	0.03–0.53
	Completed suicide	0/20*	23,189/24,378	0.03	0.00–0.43
Naltrexone/bupropion (Contrave®)	Suicidal ideation	73/135	5910/41,615	3.84	2.89–5.11
	Suicide attempt	7/12	5910/41,615	4.11	1.62–10.45
	Completed suicide	1/19	5910/41,615	0.37	0.05–2.77
Phentermine/topiramate (Qsymia®)	Suicidal ideation	34/174	5270/42,246	1.57	1.09–2.27
	Suicide attempt	4/15	5270/42,246	2.14	0.71–6.45
	Completed suicide	0/20*	5270/42,246	0.20	0.01–3.32

Counts correspond to the number of suicidality-related reports (n) compared with all other events for the same drug. Disproportionality estimates were calculated using the Haldane–Anscombe correction when needed. No statistically significant disproportionality signals were detected for suicidal ideation or suicide attempt with GLP-1–based agents. A statistically significant estimate for completed suicide was observed for liraglutide and is interpreted as statistically fragile and hypothesis-generating.

ROR, *reporting odds ratio*; AE, *adverse event*

For semaglutide, the Reporting Odds Ratios (RORs) were 1.39 (95% CI, 0.99–1.94) for suicidal ideation, 1.38 (0.46–4.15) for suicide attempt, and 1.72 (0.62–4.74) for completed suicide, none reaching statistical significance. Liraglutide displayed a comparable ROR for suicidal ideation (1.01; 0.66–1.55) and suicide attempt (0.91; 0.21–3.94), but a markedly higher signal for completed suicide (18.11; 6.96–47.15), based on a small number of cases. In contrast, tirzepatide showed consistently low disproportionality estimates: 0.19 (0.13–0.28) for suicidal ideation, 0.12 (0.03–0.53) for suicide attempt, and 0.03 (0.00–0.43) for completed suicide—all well below unity, suggesting no safety signal. These low values should be interpreted with caution, as tirzepatide’s short post-marketing history and comparatively small number of FAERS reports result in very wide confidence intervals and likely under-ascertainment of rare events. Among non-incretin combinations, naltrexone/bupropion exhibited elevated RORs for suicidal ideation (3.84; 2.89–5.12) and suicide attempt (4.11; 1.62–10.45), whereas phentermine/topiramate showed a moderate increase for suicidal ideation (1.57; 1.09–2.27) but not statistically significant results for suicide attempt or completed suicide.

At the class level, GLP-1 receptor agonists and dual incretin therapies showed disproportionality estimates below unity for suicidal ideation (ROR 0.29; 95% CI 0.22–0.38) and suicide attempt (ROR 0.22; 95% CI 0.09–0.56), but a not statistically significant elevation for completed suicide (ROR 5.85; 95% CI 0.78–43.67). In contrast, non-GLP-1 anti-obesity drugs exhibited higher reporting for suicidal ideation (ROR 3.50; 95% CI 2.67–4.61) and suicide attempt (ROR 4.48; 95% CI 1.80–11.13), while completed suicides remained infrequent (ROR 0.17; 95% CI 0.02–1.28) (Table 2).

**Table 2:** Comparative disproportionality analysis at the class level between GLP-1/dual incretin and non-GLP-1 anti-obesity drugs

		Drug cases/total cases	Drug AE reports/total AE reports	ROR	95%CI
GLP-1 (Semaglutide, Liraglutide, Tirzepatide)	Suicidal ideation	100/107	36,358/11,180	0.29	0.22–0.38
	Suicide attempt	8/11	36,358/11,180	0.22	0.09–0.56
	Completed suicide	19/1	36,358/11,180	5.85	0.78–43.67
Other non-GLP-1 (Naltrexone/bupropion, Phentermine/topiramate)	Suicidal ideation	107/100	11,180/36,358	3.50	2.67–4.61
	Suicide attempt	11/8	11,180/36,358	4.48	1.80–11.13
	Completed suicide	1/19	11,180/36,358	0.17	0.02–1.28

GLP-1 receptor agonists and dual incretin therapies as a group showed RORs below unity for suicidal ideation and suicide attempt, with a non-significant elevation for completed suicide. Conversely, non-GLP-1 combinations exhibited higher RORs for ideation and attempts. Class-level estimates should be interpreted as complementary and hypothesis-generating, as they may mask heterogeneity between individual agents.

ROR, *reporting odds ratio*; AE, *adverse event*

**Fig. 1:**
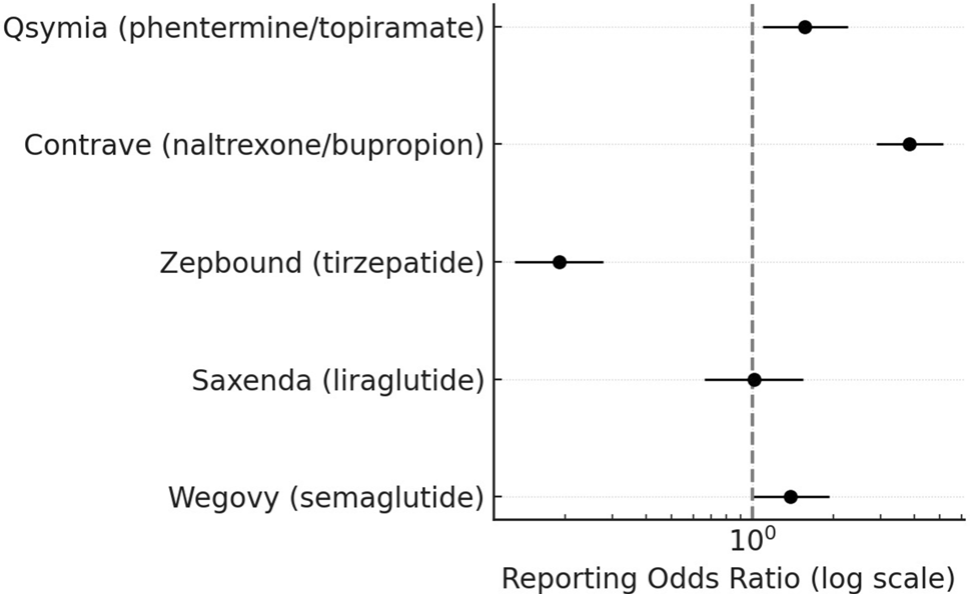
Suicidal ideation reports associated with anti-obesity drugs in FAERS (2012–2025). Forest plot showing reporting odds ratios (log scale) with 95% confidence intervals for suicidal ideation by drug. No GLP-1–based agent exceeded the null value (ROR = 1)

**Fig. 2:**
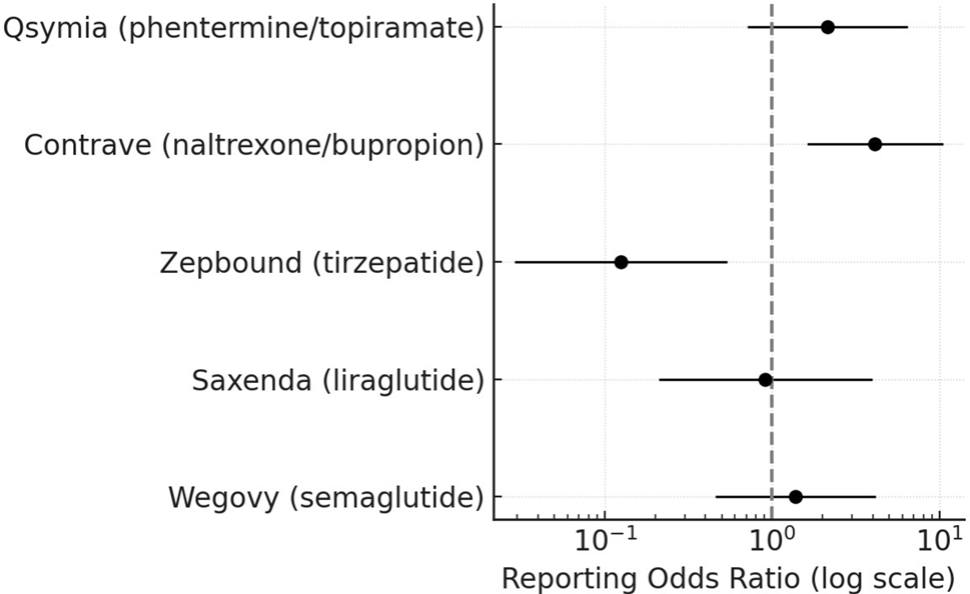
Suicide attempt reports associated with anti-obesity drugs in FAERS (2012–2025). Forest plot displaying reporting odds ratios (log scale) and 95% confidence intervals for suicide attempts. Only non-GLP-1 combinations (naltrexone/bupropion and phentermine/topiramate) showed elevated but variable RORs

**Fig. 3:**
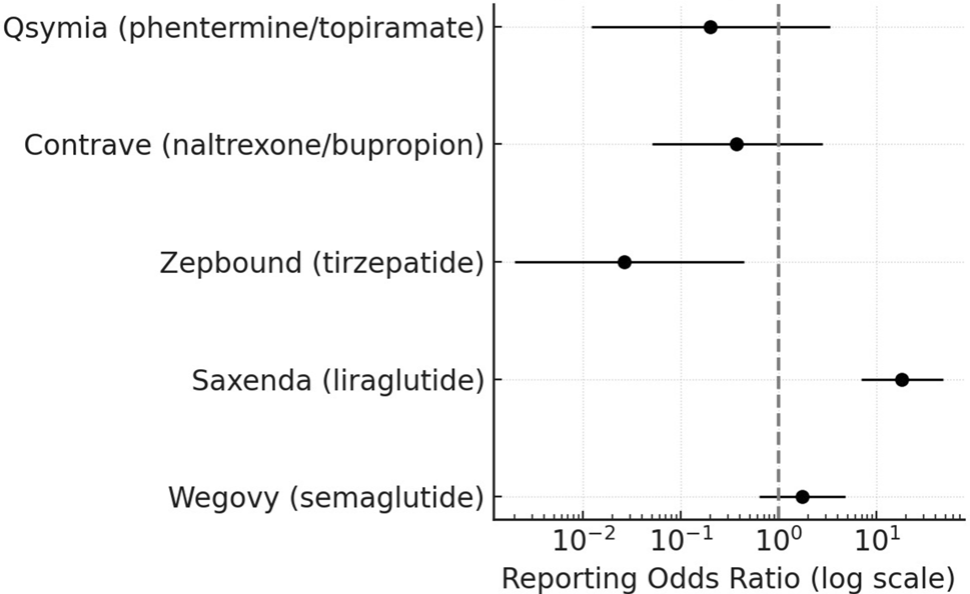
Completed suicide reports associated with anti-obesity drugs in FAERS (2012–2025). Liraglutide exhibited a numerically elevated ROR based on a small number of reports, whereas other incretin and non-incretin agents showed no signal. The log scale allows visualization of wide confidence intervals

**Fig. 4:**
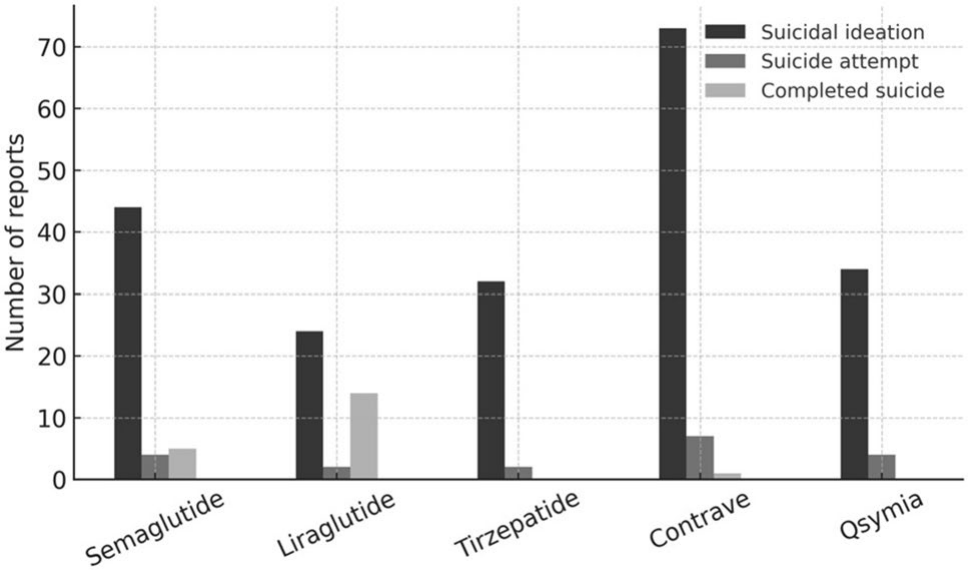
Number of FAERS reports included per anti-obesity drug (2012–2025). Bar chart summarizing total FAERS report volume per drug, illustrating differences in post-marketing exposure across agents

Figure 1 displays RORs for suicidal ideation, Fig. 2 for suicide attempt, and Fig. 3 for completed suicide. Table 2 shows class-level comparisons between GLP-1/dual incretin and non-GLP-1 agents, while Fig. 4 illustrates the overall number of FAERS reports included in the analysis.

## Discussion

The present pharmacovigilance analysis did not identify disproportionality signals for suicidal ideation or suicide attempt with any GLP-1–based anti-obesity drug. For completed suicide, a statistically significant disproportionality signal was detected for liraglutide (ROR 18.1; 95% CI 6.96–47.15), although this estimate was derived from a very small number of cases and is likely subject to statistical instability and reporting biases. Semaglutide and tirzepatide showed no elevation in reporting for any suicidality-related outcome, and class-level analyses indicated lower reporting frequencies for incretin versus non-GLP-1 therapies. It is important to emphasize that disproportionality measures such as the ROR do not estimate incidence or causality but rather detect deviations in reporting patterns within a spontaneous reporting system. Accordingly, the observed estimates should be interpreted as pharmacovigilance signals rather than indicators of clinical risk.

### Comparison with previous pharmacovigilance and clinical evidence

Our findings align with recent pharmacovigilance investigations conducted using both FAERS and WHO VigiBase® database. McIntyre et al. (2025) analyzed over 25,000 reports from VigiBase and confirmed that no causal link could be established between GLP-1receptor receptor agonists and suicidality [13]. Similarly, Wang et al. (2025) and Guirguis et al. (2024) reported that while semaglutide displayed isolated numerical elevations in the reporting of depression or self-injury terms, these signals disappeared after adjustment for concomitant medications and indication [11, 12].

A U.S. Food and Drug Administration review (2024) and the European Medicines Agency (EMA) Pharmacovigilance Risk Assessment Committee also concluded that a causal relationship was unlikely, despite the increased media attention in 2023 [38, 39].

In real-world evidence studies, including large retrospective cohorts and electronic health record analyses, most reports found no increase in suicidal ideation or behavior among patients treated with GLP-1 receptor agonists compared with other antidiabetic agents. However, one exploratory pharmacovigilance study detected preliminary suicide-related signals for semaglutide in weight-loss populations, warranting further confirmation in large prospective datasets [27–29].

Data from randomized controlled trials also consistently demonstrates the absence of psychiatric safety signals, both in diabetes and obesity populations [3, 30, 31].

For instance, pooled analyses from the PIONEER and SUSTAIN programs found that rates of depression, anxiety, or suicidality were comparable to placebo, reinforcing the biological plausibility of a null association [32].

### Possible explanations for liraglutide findings

The 2023–2024 regulatory reviews and extensive media coverage of GLP-1 receptor agonists and suicidality are also likely to have contributed to notoriety bias. In our dataset, suicidality-related reports were concentrated in the most recent years, coinciding with the rapid uptake of incretin-based anti-obesity therapies and the period of heightened public and regulatory attention. Such stimulated reporting may inflate disproportionality estimates for older or more widely used drugs, while underestimating potential signals for newer agents with shorter post-marketing histories.

Interpretation of the elevated ROR for completed suicide with liraglutide requires particular caution. The 2023–2024 regulatory reviews and extensive media attention regarding GLP-1 receptor agonists and suicidality likely contributed to stimulated reporting and notoriety bias. Liraglutide, as one of the earliest anti-obesity incretin agents, has substantially greater cumulative post-marketing exposure than newer therapies, increasing the probability that rare events are reported disproportionately. Importantly, the absolute number of completed-suicide reports was extremely small (n = 14), generating wide and statistically unstable confidence intervals. Misclassification or heterogeneity in FAERS coding may further inflate this estimate. Taken together, these considerations indicate that the observed signal is statistically fragile and should be regarded as hypothesis-generating, requiring cautious interpretation rather than being taken as definitive evidence of a pharmacological effect.

Given the extremely low number of completed suicide reports and the wide confidence intervals, this finding should not be interpreted as evidence of increased risk. Instead, the signal reflects statistical fragility and stimulated reporting rather than a reproducible pharmacological phenomenon.

Interpretation of tirzepatide disproportionality estimates is inherently limited by pronounced exposure and reporting imbalance. As the newest anti-obesity therapy, tirzepatide has accumulated far fewer FAERS reports than semaglutide or liraglutide, producing immature pharmacovigilance data with wide confidence intervals. Accordingly, the very low RORs observed for tirzepatide should not be interpreted as evidence of a protective effect, but rather as a reflection of insufficient follow-up and lower reporting volume.

It is also important to acknowledge that the potential explanations for the liraglutide signal—greater cumulative exposure, longer time on the market, and heightened regulatory/media attention—are bidirectional and cannot be used to adjudicate causality. Increased exposure may inflate reporting through stimulated reporting or selection bias, but it may equally increase the likelihood of detecting true drug-event associations. FAERS does not provide the necessary information to disentangle these possibilities. Therefore, the elevated ROR for completed suicide cannot be dismissed as non-causal on the basis of exposure history alone; rather, it represents a pharmacovigilance signal that requires cautious interpretation and external validation in controlled settings.

### Neuropharmacological considerations for non-GLP-1 anti-obesity agents

Interpretation of class comparisons requires careful consideration of the distinct neuropharmacological profiles of non-GLP-1 anti-obesity drugs. Naltrexone/bupropion combines an opioid antagonist with a dopaminergic–noradrenergic reuptake inhibitor, a mechanism known to modulate mood, reward processing, and impulsivity [16]. Bupropion-containing regimens have been associated with neuropsychiatric adverse events in both clinical trials and post-marketing settings, which may partly account for the markedly higher RORs observed in our analysis for suicidal ideation and suicide attempt.

Phentermine/topiramate also carries theoretical psychiatric implications due to topiramate’s associations with cognitive slowing, mood changes, and irritability [17]. These pharmacodynamic effects differ fundamentally from the incretin-based mechanism of GLP-1 and dual GIP/GLP-1 agonists, which do not engage monoaminergic pathways linked to suicidality.

As a result, the higher disproportionality detected among non-GLP-1 agents may reflect drug-specific neuropharmacology and differences in baseline psychiatric risk rather than evidence of a class-wide safety contrast [35–37].

### Class-level interpretation considerations

Because suicidality-related events were rare, a class-level comparison between GLP-1/dual incretin and non-GLP-1 therapies was included as a complementary analysis. However, this aggregation must be interpreted with caution. Combining semaglutide, liraglutide and tirzepatide into a single category may conceal substantial heterogeneity in post-marketing exposure, reporting volume, and time on the market. Class-level RORs therefore do not indicate a protective effect of the incretin class; rather, they underscore the importance of evaluating each active substance individually, as class aggregation can dilute or obscure drug-specific patterns. This principle is consistent with established pharmacovigilance methodology—where analyses are ideally conducted at the active-substance level—and reinforces that class-level estimates are hypothesis-generating and should not guide clinical.

### Broader context and mechanistic considerations

Although several mechanistic hypotheses have proposed links between GLP-1 signalling and central dopaminergic or stress-related pathways, preclinical findings remain inconsistent [33, 34]. Some models describe modulation of stress reactivity or mood, whereas others suggest anxiolytic rather than pro-depressive effects. Clinical studies assessing neuropsychiatric outcomes—such as cognition, addiction-related behaviour, or appetite regulation—have likewise shown neutral or beneficial central effects of GLP-1 receptor agonists [35, 36].

The particularly low RORs observed for tirzepatide, including a near-zero estimate for completed suicide, likely reflect its recent market authorization and limited post-marketing exposure time. Zepbound® was only approved for obesity in late 2023, resulting in fewer cumulative reports relative to older GLP-1 agents such as liraglutide and semaglutide. Hence, these data may underestimate rare events due to incomplete pharmacovigilance accrual rather than true absence of risk. The emerging evidence on tirzepatide, a dual GIP/GLP-1 receptor agonist, also supports a neutral psychiatric profile, consistent with our findings [37].

### Contextualisation with historical pharmacovigilance data

To contextualise our findings, it is important to consider the relative frequency of suicidality-related reports historically observed across anti-obesity drug classes. Non-GLP-1 agents, particularly naltrexone/bupropion and phentermine/topiramate, have consistently generated a higher proportion of neuropsychiatric adverse event reports in both FAERS and other international pharmacovigilance repositories [12, 16, 17]. In contrast, incretin-based therapies have historically shown low reporting rates for suicidality-related Preferred Terms, with multiple FAERS, VigiBase and real-world cohort analyses reporting either no disproportionality signal or numerically lower rates compared with active comparators [13, 14, 27–29]. These historical patterns are consistent with the class differences observed in our dataset, suggesting that the relatively higher RORs among non-GLP-1 drugs reflect established pharmacovigilance trends rather than new or unexpected safety concerns. However, interpretation should remain cautious, as reporting frequencies may also be shaped by drug utilisation patterns, media attention, and evolving regulatory scrutiny over time.

### Regulatory and public perception aspects

The temporary EMA investigation in 2023, triggered by spontaneous reports of suicidal thoughts among GLP-1 users, likely contributed to reporting amplification rather than true incidence changes.

Both the EMA and FDA concluded that available data did not support label modifications or additional warnings [38, 39].

### Implications and limitations

These findings reinforce the overall psychiatric safety of modern anti-obesity pharmacotherapy, particularly incretin-based therapies. While sporadic reports of self-harm or mood alterations continue to emerge, their absolute frequency remains extremely low compared with global exposure.

Confounding by indication remains a major limitation, as obesity and related comorbidities inherently increase baseline risks of depression and suicidality. FAERS lacks reliable information on psychiatric history, depression, socioeconomic status, substance use, and other determinants of suicidality; therefore, no adjustment for these factors was possible.

Confounding by indication is likely an important contributor to any disproportionality signal, particularly for liraglutide. Although we explored the feasibility of stratification using concomitant antidepressant or antipsychotic medications as proxy indicators of psychiatric burden, FAERS coding of concomitant therapies is incomplete, inconsistently structured, and affected by substantial missingness. As a result, no valid stratified disproportionality analysis could be performed.

Although we considered stratifying RORs by calendar period, the extremely low number of suicidality-related events—particularly completed suicides—produced highly unstable and non-interpretable time-specific ROR estimates. For this reason, we did not conduct formal temporal disproportionality modelling. References to notoriety bias in the Discussion therefore reflect conceptual mechanisms well described in spontaneous reporting systems during periods of regulatory or media attention, rather than empirical temporal testing.

Several structural limitations inherent to spontaneous reporting systems must also be acknowledged, including under-reporting, the absence of exposure denominators, and regional variability in reporting patterns. Restricting analyses to U.S. submissions and to primary suspect drugs may affect comparability with studies using broader definitions; however, these methodological choices also reduce heterogeneity and misattribution, improving internal consistency.

Misclassification is possible, particularly for completed suicide, a rare and heterogeneously coded event for which narrative details are often unavailable. Variable report quality, incomplete narratives, and inconsistent coding can further contribute to uncertainty.

Finally, additional forms of reporting bias—including competition bias, channeling bias, and stimulated reporting following regulatory or media attention—may influence the distribution of Preferred Terms across drug classes. For these reasons, FAERS-based disproportionality estimates should be interpreted cautiously and regarded as hypothesis-generating rather than evidence of causal risk.

interpretation.

## Conclusion

In this FAERS analysis, GLP-1 receptor agonists and dual incretin therapies showed no disproportionality signal for suicidal ideation or suicide attempt. A statistically significant disproportionality estimate for completed suicide was observed for liraglutide, but the very small number of cases and wide confidence intervals indicate substantial statistical fragility. These findings collectively support a neutral psychiatric safety profile for incretin-based therapies while highlighting the importance of continued pharmacovigilance, particularly for rare events such as completed suicide.

## Supplementary Information

Below is the link to the electronic supplementary material.

Supplementary file1 (DOCX 14 kb)

## Data Availability

All data analyzed in this study are publicly accessible through the openFDA platform (https://open.fda.gov/data/faers/).
