# Supplementary material for: Comparative pharmacovigilance analysis of suicidality-related adverse events among GLP-1 and non-GLP-1 anti-obesity drugs in the FDA Adverse Event Reporting System
Source: Int J Clin Pharm. 2026 Feb 25;48(3):1026–35. doi: 10.1007/s11096-026-02099-y (PMC13176177; doi:10.1007/s11096-026-02099-y)
Supplement: Supplementary file 1 — Supplementary file1 (DOCX 14 kb) [file 11096_2026_2099_MOESM1_ESM.docx]

**Supplementary Table S1. MedDRA Preferred Terms (PTs) used to identify suicidality-related events in FAERS, with representative Lowest Level Terms (LLTs)**

| **Preferred Term (PT)** | **Representative LLTs included under this PT** |
| --- | --- |
| **Suicidal ideation** | Suicidal ideation; Thoughts of self-harm; Suicidal thoughts; Morbid thoughts; Ideation suicidal |
| **Suicide attempt** | Suicide attempt; Attempted suicide; Failed suicide attempt; Self-harm attempt; Intentional self-injury |
| **Completed suicide** | Completed suicide; Suicide; Death by suicide; Self-inflicted death; Intentional self-harm fatal |

Note: All FAERS records mapped to these PTs were included, irrespective of the underlying LLT. The LLTs shown are illustrative examples, not the complete set of MedDRA terms.
